# Coding-Complete Genome Sequence of SARS-CoV-2 Isolate from Bangladesh by Sanger Sequencing

**DOI:** 10.1128/MRA.00626-20

**Published:** 2020-07-09

**Authors:** M. Moniruzzaman, Mohammad Uzzal Hossain, M. Nazrul Islam, M. Hadisur Rahman, Irfan Ahmed, Tahia Anan Rahman, Arittra Bhattacharjee, M. Ruhul Amin, Asif Rashed, Chaman Ara Keya, Keshob Chandra Das, M. Salimullah

**Affiliations:** a Molecular Biotechnology Division, National Institute of Biotechnology, Dhaka, Bangladesh; b Bioinformatics Division, National Institute of Biotechnology, Savar, Dhaka, Bangladesh; c Plant Biotechnology Division, National Institute of Biotechnology, Savar, Dhaka, Bangladesh; d Department of Biochemistry and Microbiology, North South University, Bashundhara, Dhaka, Bangladesh; e Center for Medical Biotechnology, MIS, Directorate General of Health Services, Dhaka, Bangladesh; f Department of Microbiology, Mugda Medical College, Dhaka, Bangladesh; Queens College

## Abstract

A coding-complete genome sequence of a severe acute respiratory syndrome coronavirus 2 (SARS-CoV-2) isolate was revealed. The sample for the virus was isolated from a female patient from Dhaka, Bangladesh, suffering from coronavirus disease-2019 (COVID-19).

## ANNOUNCEMENT

Severe acute respiratory syndrome coronavirus 2 (SARS-CoV-2), a member of the *Coronaviridae* family and *Betacoronaviru*s genus, is the causative agent of pandemic coronavirus disease-2019 (COVID-19). In Bangladesh, the rate of positive cases and the death toll from COVID-19 are increasing at an alarming rate (https://corona.gov.bd/). To understand the genomic characteristics of SARS-CoV-2 in Bangladesh, several isolates have been sequenced and deposited in GISAID (https://www.gisaid.org/). However, those isolates have been sequenced using a next-generation sequencing platform, except for the one we are reporting. In this study, we sequenced the viral genome by Sanger sequencing technology, which is a gold standard method and is necessary for thorough genomic analysis (1).

The isolate (SARS-CoV-2/human/BGD/NIB_01/2020) was collected from an oropharyngeal specimen on 11 May 2020. The patient was a 28-year-old saleswoman who tested positive (via reverse transcriptase PCR [RT-PCR]) for COVID-19 with symptoms of cough, mild fever, and throat congestion. (all applicable international, national, and/or institutional guidelines for the care and use of animals were followed; ethical approval number NIBREC2020-01). The viral RNA was extracted directly from the patient’s specimen using the PureLink viral RNA/DNA minikit (Invitrogen). The viral RNA was then converted into cDNA using a SuperScript VILO cDNA synthesis kit (Invitrogen).

To cover the whole genome of the virus, 48 pairs of primers were designed by following two conditions: (i) their sequence is conserved among all the available SARS-CoV-2 isolates, and (ii) the terminal of the amplicons will overlap the adjacent amplicon (Table 1). These primers underwent PCR and generated 96 amplicons, which were visualized using 1.5% agarose gel electrophoresis. The PCR products were then purified using the PureLink PCR purification kit (Thermo Fisher Scientific, USA). These purified amplicons were finally sequenced with 2× coverage using the Sanger dideoxy method by “ABI 3500” with a BigDye Terminator version 3.1 cycle sequencing kit (Applied Biosystems, USA).

**TABLE 1:** Information about primers, amplicon size, and overlapping length

Amplicon	Primer	Sequence	Product size (bp)	Overlapping length (bp)
1	Forward	AGGTTTATACCTTCCCAGG	765	131
1	Reverse	CACCACTGCTATGTTTAGTG		
2	Forward	CGCAAGGTTCTTCTTCGTA	797	
2	Reverse	AGACTATGCTCAGGTCCTAC		100
3	Forward	AAGAAGGTGCCACTACTTG	794	
3	Reverse	GTTAGTTAGCCACTGCGAA		114
4	Forward	ACTGAGACTCATTGATGCTA	788	
4	Reverse	TCACACTCTTGTAACCTTGC		130
5	Forward	AGAAAAGTACTGTGCCCTTG	783	
5	Reverse	ACCACTGTTGGTTTTACCTT		131
6	Forward	GATGGAACTTACACCAGTTG	781	
6	Reverse	GTCACTAACAAGAGTGGCAG		110
7	Forward	GAAGAAGTTACAACAACTCTGG	718	
7	Reverse	AAACTGTAGCTGGCACTTTG		136
8	Forward	TGTAGCGTCACTTATCAACA	746	
8	Reverse	CAGTGGCAAGATAACAGTTG		158
9	Forward	CTCTACGTGTTGAGGCTTTT	720	
9	Reverse	CATCCGTAATAGGACCTTTGT		130
10	Forward	TTGTGCTAGTGAGTACACTG	760	
10	Reverse	AATGTCTCCTACAACTTCGG		150
11	Forward	TGATGTACTGAAGTCAGAGG	737	
11	Reverse	AATAGCCTTCTCTGTAACCAG		90
12	Forward	TTCTTTAATCTACTCAACCGC	706	
12	Reverse	CTGTAGTGACAAGTCTCTCG		118
13	Forward	ATGCTAATGGAGGTAAAGGC	701	
13	Reverse	ACAACTATCGCCAGTAACTTC		115
14	Forward	CTTTTATTTCAGCAGCTCGG	714	
14	Reverse	GTGCGTAATATCGTGCCA		133
15	Forward	GCTGATTTTGACACATGGTT	812	
15	Reverse	GGTAAGAATGAGTAAACTGGTG		196
16	Forward	CCTATTGGTGCTTTGGACATA	727	
16	Reverse	AACCCTCAACTTTACCAGATG		146
17	Forward	CTTGTTGTCATCTCGCAAAG	767	
17	Reverse	TCGATTGAGAAACCACCTGT		112
18	Forward	TTGTTGACAGGCAAACAGC	770	
18	Reverse	ACCATCATCATACACAGTTCT		121
19	Forward	TGACATGGTTGGATATGGTTG	794	
19	Reverse	GTTTATGTCTACAGCACCCT		172
20	Forward	AATTGTGGGCTCAATGTGT	787	
20	Reverse	GCAACAGGACTAAGCTCATTA		155
21	Forward	GGAAATCCAACAGGTTGTAGA	795	
21	Reverse	ACAGGGTCATTAGCACAAGT		90
22	Forward	GTTGCCACATAGATCATCCAA	790	
22	Reverse	AACAATACCAGCATTTCGC		233
23	Forward	GCAGACCTCGTCTATGCTTT	813	
23	Reverse	GCACGTAGTGCGTTTATCT		147
24	Forward	CCACTTCAGAGAGCTAGGTG	782	
24	Reverse	GTGAGGGTTTTCTACATCACT		114
25	Forward	ATTGAAATCAATAGCCGCCA	775	
25	Reverse	ATCTGGGTAAGGAAGGTACA		117
26	Forward	GTCTGAAGCAAAATGTTGGA	805	
26	Reverse	GAGTCTTTCAGTACAGGTGTT		142
27	Forward	TGTGTGCTAATGGACAAGTT	784	
27	Reverse	TCAAAACACTCTACACGAGC		132
28	Forward	CTTCTGCTCGCATAGTGTAT	769	
28	Reverse	CAAGAGTGAGCTGTTTCAGT		191
29	Forward	AATAGGCGTGGTAAGAGAAT	790	
29	Reverse	GTACATAAGTGGTATGAGGTGT		139
30	Forward	AGCTAGGTTTTTCTACAGGTG	756	
30	Reverse	CTTTGTCACTACAAGGCTGT		152
31	Forward	GTAGAAAGGTTCAACACATGG	733	
31	Reverse	ATAGAAACTGGTACTTCACCC		144
32	Forward	GCTTTAGCTTGTGGGTTTAC	808	
32	Reverse	CCACCTAACTGACTATGACT		139
33	Forward	CAAGAATTTAAACCCAGGAG	758	
33	Reverse	GCATCAGAGACAAAGTCATT		155
34	Forward	CACATTAACATTAGCTGTACCC	781	
34	Reverse	TGACTAGAGACTAGTGGCA		182
35	Forward	AAGGGGTACTGCTGTTATGT	775	
35	Reverse	TTAATAGGCGTGTGCTTAGA		116
36	Forward	TCAGCCTTTTCTTATGGACC	794	
36	Reverse	TCCAAGCTATAACGCAGC		104
37	Forward	TTAGAGGTGATGAAGTCAGA	760	
37	Reverse	TGTTCAGCCCCTATTAAACA		149
38	Forward	TAACCAGGTTGCTGTTCTTT	797	
38	Reverse	CAATCATTTCATCTGTGAGCA		191
39	Forward	CAGATCCATCAAAACCAAGC	771	
39	Reverse	GCAAGAAGACTACACCATGA		137
40	Forward	TCAGAGCTTCTGCTAATCTTG	759	
40	Reverse	GTAATTTGACTCCTTTGAGC		137
41	Forward	TTGCCATAGTAATGGTGACA	798	
41	Reverse	AGCTGGTAATAGTCTGAAGTG		120
42	Forward	GCACAACAAGTCCTATTTCT	784	
42	Reverse	CCATAACAGCCAGAGGAAAA		170
43	Forward	GCAGATTCCAACGGTACT	707	
43	Reverse	TAGTAACCTGAAAGTCAACG		117
44	Forward	GCTACAGGATTGGCAACTAT	785	
44	Reverse	TTTCATGTTCGTTTAGGCGT		174
45	Forward	CACTTTGCTTCACACTCAAA	791	
45	Reverse	TCTGGACTGCTATTGGTGTT		180
46	Forward	CAGATTCAACTGGCAGTAAC	793	
46	Reverse	TTTCCTTGGGTTTGTTCTGG		187
47	Forward	CTGCTTGACAGATTGAACCA	698	
47	Reverse	CTTGTGCTATGTAGTTACGAGA		242
48	Forward	ATGAAACTCAAGCCTTACCG	518	
48	Reverse	CCTTTCGTGCAGGTCAATA		

The raw reads were assembled using DNA Sequence Assembler version 4 (2013) (Heracle BioSoft) and verified with SeqMan Pro version 14.1 (DNAStar, Madison, WI). After assembly, 48 contigs with 94 overlapping regions were obtained. These overlapping regions were visualized using CLC Genomics Workbench version 20.0.4 and merged with EMBOSS: merger (2).

The assembled viral genome consists of a single-stranded positive (+) RNA that is 29,724 nucleotides long. The NCBI BLASTN program (3) showed that the genome was mostly similar to SARS-CoV-2/human/BGD/CHRF_0001/2020 (GenBank accession number MT476385.1). From NCBI, the FASTA sequences of 7 mostly similar genomes from Bangladesh, India, Sri Lanka, and the United States were taken along with the reference genome. Another 16 genomes of SARS-CoV-2 that were isolated in Bangladesh were collected from GISAID (https://www.gisaid.org/). The genomes were aligned with MAFFT version 7 using default parameters (4). The phylogenetic tree was constructed using FastTree version 2.1.10 (5) through the Galaxy platform (6). Here, the tree was built by nucleotide alignment using the generalized time-reversable model (GTR) plus the CAT nucleotide evolution model (GTR+CAT). The tree was visualized using iTOL (7), where the tree structure was rerooted on the position of reference isolate SARS-CoV-2 Wuhan-Hu-1.

**FIG 1:**
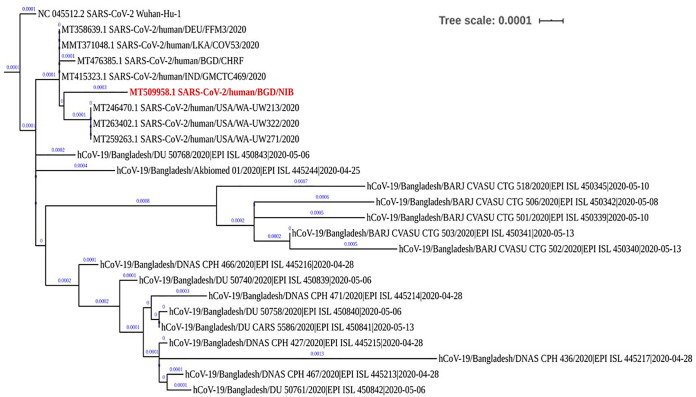
Phylogenetic analysis of the SARS-CoV-2/human/BGD/NIB_01/2020 isolate. Nucleotide alignment and the GTR+CAT nucleotide evolution model was applied to construct the tree. The tree was visualized using i-TOL. Here, the *x* axis represents the tree scale. A scale bar with a 0.0001 value is given on the top. The genome (labeled in red) shares a common ancestor with some isolates from the United States.

The genome has 8 nucleotide differences from the closest isolate. Interestingly, except for isolate SARS-CoV-2/human/BGD/CHRF0001/2020, the other strains of SARS-CoV-2 from Bangladesh showed separate clades and distant genetic relations. The tree also demonstrated that our viral genome and three isolates from the United States share an ancestor (Fig. 1).

### Data availability.

The complete nucleotide sequence of this SARS-CoV-2 isolate (SARS-CoV-2/human/BGD/NIB_01/2020) has been deposited in GenBank under the accession number MT509958.
